# A versatile microfluidic platform measures hyphal interactions between *Fusarium graminearum* and *Clonostachys rosea* in real-time

**DOI:** 10.1038/s42003-021-01767-1

**Published:** 2021-02-26

**Authors:** Alejandro Gimeno, Claire E. Stanley, Zacharie Ngamenie, Ming-Hui Hsung, Florian Walder, Stefanie S. Schmieder, Saskia Bindschedler, Pilar Junier, Beat Keller, Susanne Vogelgsang

**Affiliations:** 1 Ecological Plant Protection in Arable Crops, Plant Protection, Agroscope, Zurich, Switzerland; 2 Molecular Plant Biology and Phytopathology, Department of Plant and Microbial Biology, University of Zurich, Zurich, Switzerland; 3 Institute for Chemical and Bioengineering, ETH Zürich, Zürich, Switzerland; 4 Plant-Soil Interactions, Agroecology and Environment Research Division, Agroscope, Zurich, Switzerland; 5 Institute of Microbiology, Department of Biology, ETH Zurich, Zurich, Switzerland; 6 Laboratory of Microbiology, University of Neuchâtel, Neuchâtel, Switzerland; 7 Present Address: Department of Bioengineering, Imperial College London, London, UK; 8 Present Address: Division of Gastroenterology, Boston Children’s Hospital, Harvard Medical School, Boston, MA USA

**Keywords:** Lab-on-a-chip, Fungal ecology, Agriculture

## Abstract

Routinely, fungal–fungal interactions (FFI) are studied on agar surfaces. However, this format restricts high-resolution dynamic imaging. To gain experimental access to FFI at the hyphal level in real-time, we developed a microfluidic platform, a FFI device. This device utilises microchannel geometry to enhance the visibility of hyphal growth and provides control channels to allow comparisons between localised and systemic effects. We demonstrate its function by investigating the FFI between the biological control agent (BCA) *Clonostachys rosea* and the plant pathogen *Fusarium graminearum*. Microscope image analyses confirm the inhibitory effect of the necrotrophic BCA and we show that a loss of fluorescence in parasitised hyphae of GFP-tagged *F. graminearum* coincides with the detection of GFP in mycelium of *C. rosea*. The versatility of our device to operate under both water-saturated and nutrient-rich as well as dry and nutrient-deficient conditions, coupled with its spatio-temporal output, opens new opportunities to study relationships between fungi.

## Abstract (web summary)

Here, the authors developed a microfluidic platform that measures fungal-fungal interactions at the hyphal level in real-time. This device uses microchannel geometry to enhance the visibility of hyphal growth and provides control channels to allow comparisons between localized and systemic effects, which facilitated the investigation of the relationship between the noxious fungal pathogen *Fusarium graminearum* and the biological control agent *Clonostachys rosea*.

## Introduction

Fungi are ubiquitous organisms in terrestrial ecosystems and the ‘mycobiome’ fulfils an important role in the decomposition of organic and inorganic matter as well as in symbiotic (mutualistic, commensal, or pathogenic) relationships with animals and plants^1^. In order to understand the function of these mycobiomes, it is not only important to decipher the composition of fungal communities, but also to increase our understanding about the complex biological interactions between fungal species. In fact, the study of fungal–fungal interactions (FFI) has fostered the development of multiple beneficial applications. Antagonistic fungi are used for biological control of pathogens in agriculture^2^, whereas fungal secondary metabolites and enzymes are exploited as natural plant growth promoters^3,4^, therapeutic agents^5^ or in the production of renewable energies^6^.

Until recently, filamentous fungi, characterised by their formation of hyphal networks, have been investigated widely on agar plates or in liquid cultures to observe their growth behaviour and genetic or metabolic responses in FFI^7–10^. Although these studies yield valuable information on the colony level, the emergence of microfluidic technology has brought new opportunities to study microbes at the cellular and intra-cellular levels. The field of microfluidics originally stemmed from the specific needs of fields such as microelectronics and the chemical sciences, where precisely engineered, micron-scale features are applied to create microfluidic devices capable of operating with low fluid volumes and on short time scales^11^. The adoption of microfluidic technologies in the biological sciences has followed these footsteps, and capitalises on the ease of manipulation at the micron-scale level, to mimic the natural habitats of bacterial^12^, mammalian^13^, plant^14^ and fungal^15^ cells. In that way, studies on filamentous fungi using confined microfluidic structures, often referred to as ‘microchannels’, have enhanced the analysis of hyphal growth in microenvironments^16–18^. Moreover, microfluidic devices have enabled scientists to examine the physical and chemical interactions between fungi and bacteria^19^, fungi and nematodes^20,21^ or fungi and human immune cells^22^ at substantially higher temporal and spatial resolution than previously possible.

Routinely, the study of FFI on the cellular level employs the use of microscope slides coated with a thin layer of agar^23^. Alternatively, cellophane membranes are placed between the surface of agar plates and the mycelium in confrontation assays^24^, as a means to obtain samples from the hyphal–hyphal interaction zone for visualisation under a microscope at a later stage. However, due to the lack of confinement on the micron-scale and the fact that sample preparation often requires cutting and fixation of the mycelium, these classical approaches are limiting, especially concerning long-term live imaging of hyphal growth. It is a challenge to obtain quantitative information from the interaction zones. Accordingly, difficulties associated with sampling using these traditional approaches can lead to considerable variability between observations within experimental runs^25^.

Considering these limitations, we developed a microfluidic platform to explore interactions between filamentous fungi at the cellular level. The FFI device described herein allows the direct confrontation of two opposing fungi in a confined microenvironment, which contains several features to ensure the reproducibility of experiments and enable the quantification of hyphal growth using microscope image analysis. Our design incorporates micron-sized fluidic channels that act to confine and filter the hyphae, thus enhancing visibility of the hyphal exploration as well as internal control channels to facilitate the comparison of localised with systemic effects. We applied two distinct interaction scenarios between (i) the pathogenic mycotoxin producer *Fusarium graminearum* against the biological control agent (BCA) *Clonostachys rosea* and (ii) *F. graminearum* against *Trichoderma rossicum*. To demonstrate the versatility of the FFI device, we performed experiments with and without the introduction of potato dextrose broth (PDB) as aqueous nutrient source into the device, respectively. The main objective of this study was to investigate the necrotrophic hyperparasitism of *C. rosea* against *F. graminearum* at the single-cell level^26^. As a key result, we provide new evidence on this FFI with a first insight into the dynamics of a potential uptake of green fluorescence protein (GFP) by *C. rosea* from hyphae of fluorescently tagged *F. graminearum* in a time-lapse experiment.

## Results

### Design and function of the microfluidic device

**Fig. 1:**
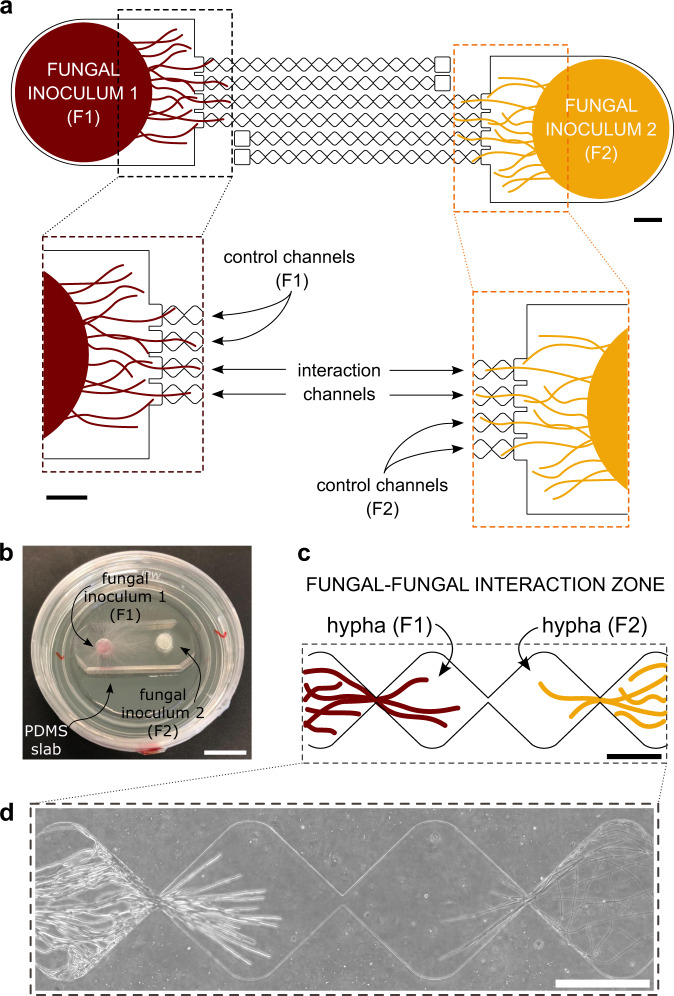
Design of the microfluidic fungal–fungal interaction (FFI) device. **a** Overview of the FFI device, showing the nature of the interaction and control channels and how they are connected to the fungal inocula. The enlarged regions of interest (dashed boxes) illustrate the directed growth of the fungal inoculum towards the control and interaction channels (duplicates of each channel type). Scale bar = 1 mm. **b** Photograph illustrating the FFI device. Two fungal inocula plugs of equal size (F1 and F2) can be introduced into the device, allowing hyphal growth and therefore confrontation of two fungal strains via interconnected microchannels. Microchannels feature on the surface of a poly(dimethylsiloxane) (PDMS) slab bonded to a glass-bottomed Petri dish. Scale bar = 1 cm. **c** Schematic representation of the interaction zone between the two fungi, consisting of equally sized, diamond-shaped segments that trigger a funnel effect and therefore regulate hyphal advance along the microchannel. Scale bar = 250 µm. **d** Phase contrast image taken from the interaction zone showing hyphae from two different fungal strains growing towards one another. Scale bar = 250 µm.

We developed the FFI device to direct and constrain the growth of two fungal cultures (F1 and F2) towards one another, making it possible to study their interaction on the cellular level (Fig. 1). The FFI device was constructed by bonding a layer of poly(dimethylsiloxane) (PDMS), having microchannels embossed into the surface at a depth of 10 µm, to a glass-bottomed Petri dish. Here, the depth of the microchannels (10 µm) accommodates the hyphae of *F. graminearum* (measured diameter of 6 ± 1 µm), while leaving sufficient room for the interaction with hyphae of *C. rosea* (3 ± 1 µm) or *Trichoderma rossicum* (3 ± 1 µm). Through bonding of the structured PDMS surface onto a sterile, glass-bottomed Petri dish, two microfluidic ‘interaction channels’ were created, spanning a length of 8.8 mm. These channels were designed to consist of 18 equally sized, diamond-shaped segments (each 490 µm × 430 µm) and thus provide a direct connection between the two fungal inlet zones (Fig. 1a, b). Importantly, the entrance to and exit of every segment is limited to 20 µm, thus effectively creating a ‘funnel’ along the microchannel and thus allowing only a few hyphae to penetrate into the following segment simultaneously (Fig. 1c). As we observed, this facilitates the study of single hyphal interactions between two growing hyphal fronts (Fig. 1d). The reduction in simultaneous hyphal penetration owing to the microchannel segmentation enables detailed observation of single hyphae over a longer period of time and helps to track morphological changes, such as branching or septation, in response to the fungal interaction. As the channels are duplicated within the design of the FFI device, the variability within the device is reduced by obtaining a mean of two measurements on the colony level.

In addition to the interaction channels, each fungal inlet zone is also connected to the ‘control channels’. These channels run in parallel to the interaction channels (also in duplicate), but consist of 16 diamond-shaped segments and therefore result in a dead end for the advancing hyphae. This way, direct contact of hyphae and of diffusible solutes secreted in the interaction with the opposing fungus is excluded, which allows to study the systemic effect of FFI through an internal control growing from the original fungal inoculum. To understand the value of the control channel, we analysed the measured growth (distance in µm) for each fungus along this channel under different conditions (Supplementary Table 1 and Supplementary Fig. 1). Statistical analyses were performed over the time period between 24 and 72 h post inoculation (hpi), from when the hyphae entered into the microchannel until the moment at which the fast-growing hyphae of *F. graminearum* reached the end of the control channel. For the experiments involving *F. graminearum* 8/1-wt-GFP vs. *C. rosea* 016 and *F. graminearum* PH1-dsRed vs. *C. rosea* 016, we found that *C. rosea* growth through the control channels was significantly slower than that of *F. graminearum* (*p* < 0.001). This is in line with the measured difference between the radial growth on agar plates after 72 h (Supplementary Fig. 2). Consistent with all experiments, we found no significant systemic effect on the growth of the fungi in the control channel by the type of interaction taking place within the adjacent interaction channel (Supplementary Table 1).

Another advantageous feature of the FFI device involves the fungal inlets that are connected to the microchannels. Homogeneous growth with high reproducibility was achieved as a result of standardising the fungal inocula to plugs of an exact size to fit within the fungal inlet comfortably and thus cover the majority of the inlet zone (Fig. 1b). The use of inlets prevents cross-contamination between the two fungal partners such as growth around the microfluidic device or uncontrolled mixing of conditioned fluids. The symmetrical alignment of the fungal inlet zones about the entrance to the microchannels avoids any bias in quantitative comparisons, as hyphae from different fungal species have exactly the same probability of entering the interaction or control channels, respectively (Fig. 1a).

During the experiments, optimal growth conditions were maintained within the sealed glass-bottomed Petri dish, providing a humid and sterile environment. The additional room around the bonded PDMS layer is sufficient to add at least 200 µl of water to maintain humid conditions. The thin cover glass bottom (0.17 ± 0.01 mm) provides excellent properties for bright field as well as fluorescence microscopy imaging (RI = 1.525).

### Differential growth rates of fungal species in interaction

**Fig. 2:**
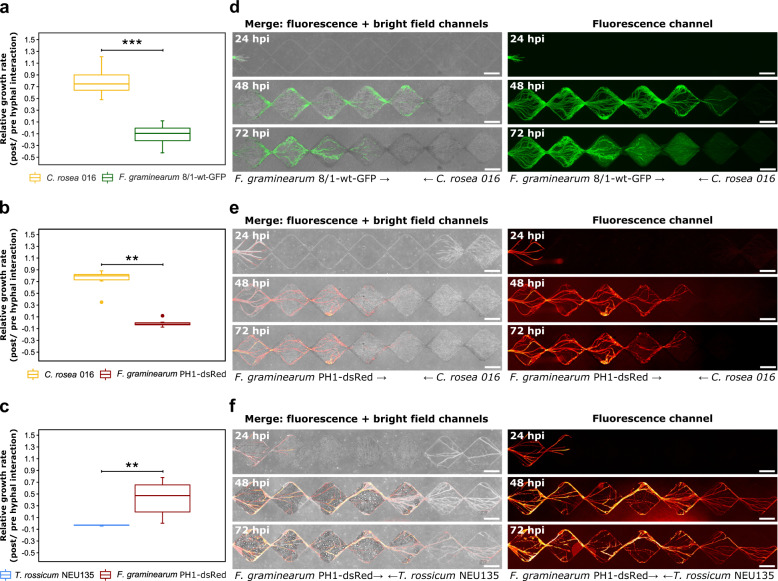
Differential growth of fungal species in the fungal–fungal interaction device. **a**–**c** Boxplots (*n* = 6) with the median (bar across box) and 25th to 75th percentiles (box) show the relative growth rate (=growth rate 24 h post hyphal interaction/growth rate 24 h pre hyphal interaction) for **a** *Clonostachys rosea* 016 vs. *Fusarium graminearum* 8/1-wt-GFP, **b** *C. rosea* 016 vs. *F. graminearum* PH1-dsRed in microchannels filled with potato dextrose broth (PDB) and **c** *Trichoderma rossicum* NEU135 vs. *F. graminearum* PH1-dsRed in dry and nutrient-deficient microchannels. Upper and lower whiskers represent the largest observation smaller or equal to the upper and lower percentile plus 1.5*interquartile range, respectively. Data points beyond the whisker range are plotted as dots. Significant differences according to the *t* test **a** or the Mann–Whitney *U* test (**b**, **c**) are indicated with asterisks for *p* values < 0.001 (***) and < 0.01(**). **d**–**f** Microscopy image sections (seven diamond segments) taken from time-lapse experiments showing interaction between fungi, specifically **d** *C. rosea* 016 vs. *F. graminearum* 8/1-wt-GFP, **e** *C. rosea* 016 vs. *F. graminearum* PH1-dsRed (microchannel filled with PDB) and **f** *T. rossicum* NEU135 vs. *F. graminearum* PH1-dsRed (dry and nutrient-deficient microchannel) after 24, 48 and 72 h post inoculation (hpi). Scale bar = 200 µm.

We applied our microfluidic device to three different FFI scenarios. The first two scenarios explored the interaction between two fluorescently tagged strains of *F. graminearum* (8/1-wt-GFP; PH1-dsRed) and the fungal antagonist, *C. rosea* strain 016. An environment saturated with PDB in the microchannels was created to ensure the availability of water and nutrients, resembling the condition that was also tested in a conventional confrontation assay on agar plates (Supplementary Fig. 3). Both interactions, i.e. between *C. rosea* 016 vs. *F. graminearum* 8/1-wt-GFP and *C. rosea* 016 vs *F. graminearum* PH1-dsRed, were analysed in time-lapse experiments. For all conditions tested, 24 hpi proved to be the most appropriate starting point to begin data collection, as both fungi had begun to enter the interaction channels (the faster growing *F. graminearum* consistently reached further into the microchannels cf. *C. rosea* 016) (Fig. 2d, e). The first growth rates were calculated after 48 hpi (24–48 hpi), when direct contact between both fungi was established. Before contact, both fungi grew steadily toward one another and no growth inhibition was observed. However, upon hyphal contact, *F. graminearum* stopped advancing along the interaction channel, whereas *C. rosea* hyphae began to enter diamond segments previously already colonised by the inhibited species *F. graminearum*. Comparisons of growth rates for *C. rosea* and *F. graminearum* hyphae 24 h before and after interaction led us to define the relative growth rate for each fungus and to confirm the significant growth-inhibiting effect of the antagonist against *F. graminearum* strains 8/1-wt-GFP (*p* < 0.001) and PH1-dsRed (*p* = 0.002), respectively (Fig. 2a, b). The changes in growth rate for each strain separately, revealed a complete inhibition of both *F. graminearum* strains, whereas *C. rosea* continued to advance within the interaction channel at reduced speed following the direct hyphal–hyphal contact (Supplementary Fig. 4a, b). Confrontation on agar plates also showed significant growth inhibition against *F. graminearum* strains 8/1-wt-GFP (*p* < 0.001) and PH1-dsRed (*p* = 0.026), respectively (Supplementary Fig. 3a, b). At this stage, the combined use of phase contrast microscopy to track *C. rosea* hyphae and fluorescence microscopy to track hyphae originating from *F. graminearum* within the FFI device proved to be highly advantageous in distinguishing the different hyphae. Strikingly, the interaction between *C. rosea* and the GFP-expressing *F. graminearum* 8/1-wt revealed an interesting phenomenon that was observed consistently. Once hyphal–hyphal contact was established, the fluorescence intensity within hyphae of *F. graminearum* 8/1-wt-GFP gradually decreased over time, whereas the background fluorescence intensity in the microchannel increased strongly (Fig. 2d). The loss of fluorescence within hyphal cells was also observed in the interaction between *C. rosea* against the dsRed-expressing *F. graminearum* PH1 strain (Supplementary Fig. 5). In the adjacent control channels within the device or in the self-interactions for *F. graminearum* 8/1-wt-GFP and *F. graminearum* PH1-dsRed, this loss of fluorescence or an increase in background fluorescence was not detected. To the contrary, over the remaining course of the experiment, fungi set up in confrontation with themselves in the FFI device resulted in the establishment of hyphal connections, presumably anastomosis (Supplementary Fig. 6a, b). Owing to the slower growth of *C. rosea*, the first hyphal contact in the device was established after 72 h in the self-interaction. Hyphae of *C. rosea* did not advance after the contact (Supplementary Fig. 6c).

The third scenario explored the interaction between *F. graminearum* PH1-dsRed and *T. rossicum* NEU135. To create a dry and nutrient-deficient environment, no PDB was introduced into the microchannels. Nevertheless, hyphae of both *T. rossicum* and *F. graminearum* were often surrounded by liquid, which was drawn into the microchannels. In parallel, control channels without fungal colonisation remained dry throughout the experiment (Supplementary Fig. 7). The formation of liquid films progressively increased the water content in the microchannels (Supplementary Movie 1). In an analogous manner to the nutrient-rich condition, the fungal hyphae grew along the microchannels at a steady pace until they came into close contact with one another. A weak growth-inhibiting effect of *T. rossicum* NEU135 against *F. graminearum* led to a reduced growth rate of the pathogen post hyphal interaction (Supplementary Fig. 4c). However, the presence of *F. graminearum* resulted in a full stop of *T. rossicum* hyphal growth (Fig. 2c and Supplementary Fig. 4c). No direct hyphal interaction between *F. graminearum* and *T. rossicum* was observed, as *F. graminearum* hyphae grew quickly and passed the areas colonised by *T. rossicum* (Fig. 2f). The comparable setup on agar plates confirmed the competitive advantage of *F. graminearum* against *T. rossicum* (Supplementary Fig. 3c). In the self-interactions for *T. rossicum* or *F. graminearum*, hyphae colonised the microchannels from both sides until they met, presumably forming hyphal connections (Supplementary Fig. 6d, e).

### Dynamics of *C. rosea* antagonising *F. graminearum*

**Fig. 3:**
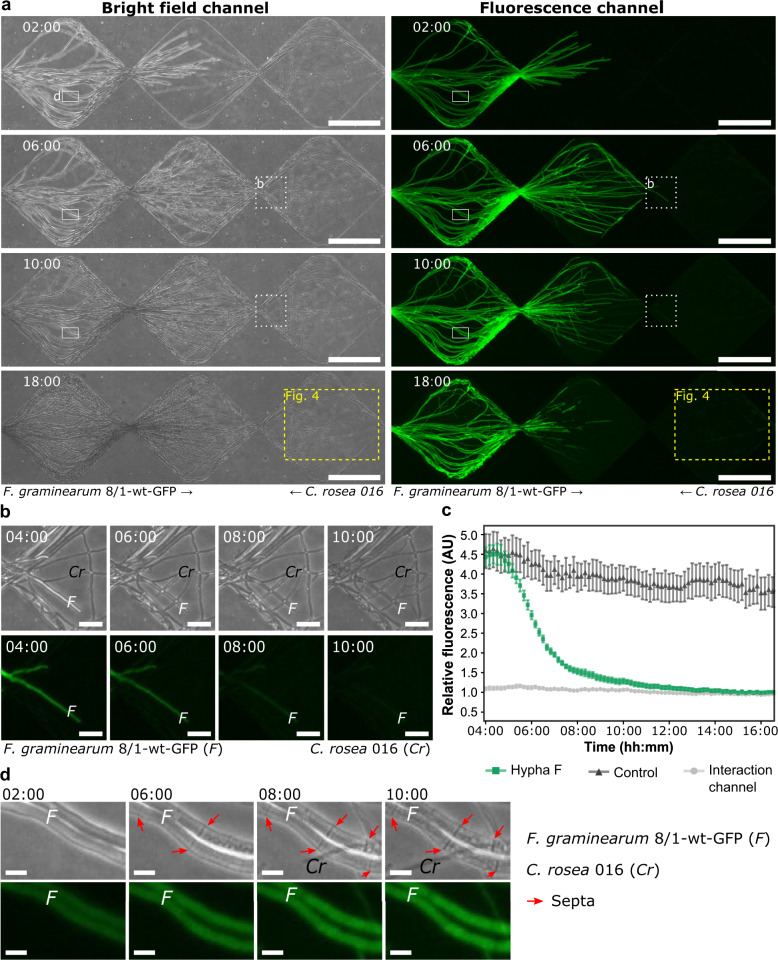
*Clonostachys rosea* 016 antagonising *Fusarium graminearum* 8/1-wt-GFP within the fungal–fungal interaction device. **a** Series of phase contrast (bright-field channel) and fluorescence microscopy images taken from a time-lapse experiment (Supplementary Movie 2) showing continued growth of *Clonostachys rosea* around the pathogen *Fusarium graminearum* and the increasing loss of the green fluorescence protein (GFP) detection from hyphae of GFP-expressing *F. graminearum*. The dashed box highlights the region of interest used for the preparation of Fig. 4. Scale bar = 200 µm. **b** Time series showing the formation of hyphal branches by *C. rosea* that form contact with *F. graminearum* before the loss of fluorescence. Scale bar = 25 µm. **c** Relative fluorescence intensities in arbitrary units (AU) over time. Error bars represent the standard error of the mean of six repetitive measurements. Hypha F =  single hypha of *F. graminearum* in contact with *C. rosea* relative to the *F. graminearum* control channel. Interaction channel = fluorescence intensity of the interaction channel relative to the *F. graminearum* control channel. Control = hyphae of *F. graminearum* not in direct contact with *C. rosea* relative to the *F. graminearum* control channel. **d** Time series showing septa formation by *F. graminearum* (red arrows) in response to hyphal contact with *C. rosea*. Scale bar = 10 µm. Time stamp format = hh:mm.

**Fig. 4:**
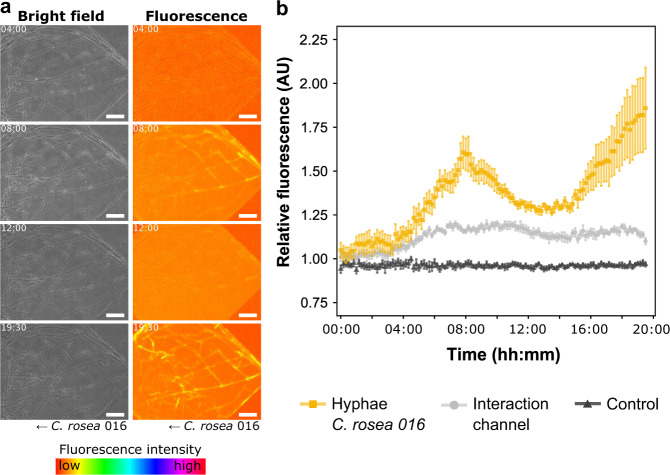
Detection of green fluorescence protein (GFP) in the hyphal network of *Clonostachys rosea* 016 antagonising *Fusarium graminearum* 8/1-wt-GFP within the fungal–fungal interaction device. **a** Series of phase contrast (bright field channel) and fluorescence microscopy images taken from a time-lapse experiment (Supplementary Movie 3) showing GFP detection in hyphae of *Clonostachys rosea* that colonised the diamond segment anterior to the fungal–fungal interaction zone. The lookup table “spectrum” (Fiji) was applied to improve the visibility of fluorescence intensity. Scale bar = 50 µm. **b** Relative fluorescence intensities in arbitrary units (AU) over time. Error bars represent the standard error of the mean of six repetitive measurements. Hyphae *C. rosea* 016 = hyphal network of *C. rosea* showing GFP detection relative to hyphae in the *C. rosea* control channel. Interaction channel = fluorescence intensity of the interaction channel relative to the *C. rosea* control channel. Control = hyphae of *C. rosea* in the control channel relative to the control channel. Time stamp format = hh:mm.

A time-lapse experiment involving *C. rosea* 016 and *F. graminearum* 8/1-wt-GFP confirmed the growth inhibition of *F. graminearum* by *C. rosea*. Moreover, we gained insights into the dynamics of mycoparasitism and the defence strategies employed by the fungus under attack by using a high temporal resolution with 10 min intervals between image acquisitions (Figs. 3 and  4).

Time-lapse image analysis started before the first hyphal contact (time point 00:00) and was conducted for 19 h and 30 min, resulting in a long-term visualisation of the antagonistic activity by *C. rosea* against *F. graminearum* at the cellular level (Supplementary Movie 2). Two hours after the first hyphal–hyphal contact, the pathogen stopped advancing along the microchannel. *C. rosea* continued growing into the pathogen-colonised areas and quickly began to form an extensive network of fine hyphal branches that made contact with the hyphae of *F. graminearum* (Fig. 3a). Along with the progressive increase of *C. rosea* hyphal branches surrounding *F. graminearum*, the measured detection of the GFP signal in the pathogen hyphae steadily decreased over time, whereas the background fluorescence within the microchannel increased (Fig. 3b, c). Concomitantly, we detected GFP fluorescence within the hyphal network of *C. rosea*. In fact, GFP was detected within the mycelium of *C. rosea* that colonised the diamond segment anterior to the FFI zone (Fig. 4a and Supplementary Fig. 8). The fluorescence intensity measured in these hyphae increased and decreased, before it finally increased again over time compared with hyphae of *C. ro*sea in the control channel (Fig. 4b). Overview images showed no fluorescence in *C. rosea* hyphae that colonised the adjacent control channels (Supplementary Fig. 8). The fusion of hyphal branches to form an interconnected network of hyphae by the antagonist as well as the detection of GFP within the hyphae of *C. rosea* is shown in Supplementary Movie 3. Furthermore, visualisation of hyphae using this high-resolution dynamic imaging technique revealed a potential defence strategy employed by *F. graminearum* when under attack. Specifically, we observed that the number of septa within hyphal cells progressively increased over time, once *C. rosea* hyphal branches grew in proximity to the pathogen (Fig. 3d).

## Discussion

Here, we present the development of a microfluidic platform for probing FFI at the cellular level. In general, devices of this kind can be used to provide mechanistic insights into a multitude of interactions between filamentous fungi relevant to the mycobiome of terrestrial and aquatic ecosystems. The application of confined and segmented microchannels allows hyphae of two fungal species to come into direct confrontation with one another, whilst ensuring directed hyphal growth and providing the opportunity to track hyphal advancement quantitatively over various time-periods using live-cell imaging. As a result, the use of a microfluidic device resolves one of the major challenges in the study of hyphal–hyphal interactions caused by the extensive hyphal branching on non-confined agar surfaces.

We obtained insights on the sequence of events during the interaction between the noxious fungal pathogen *F. graminearum* and a promising BCA, the fungal mycoparasite *C. rosea*. A deeper understanding of the mode of action of naturally occurring antagonists will help to optimise their application, which in turn contributes to their successful use in food and feed production. In fact, the use of BCAs is of increasing importance to reach the goal of reducing the negative impact of synthetic fungicides. Thanks to the optical properties of the materials used to construct our device, i.e. PDMS and glass, we obtained high-quality microscope images, whereby the combination of bright field with fluorescence microscopy delivered insights into the dynamics of the necrotrophic hyperparasitism employed by *C. rosea* against *F. graminearum*. On the cellular level, our observation clearly resembled the findings of Chatterton and Punja^27^ who employed scanning electron microscopy and performed enzyme activity assays to investigate the hyphal interaction between *C. rosea* and the soil-borne pathogen *F. oxysporum*. They identified the production of β-1,3-glucan and chitin-degrading hydrolytic enzymes by *C. rosea*, both major cell wall components in *Fusarium* species, behind the observed growth inhibition in contact between the two fungi. The observed loss of fluorescence in the hyphae of fluorophore-expressing *F. graminearum* strains upon contact with *C. rosea* is likely linked to the degradation of the cell wall of the pathogen, caused by the release of extracellular cell wall degrading enzymes (CWDEs), secreted in the presence of *Fusarium*^28,29^. Indeed, a similar phenomenon was observed in hyphae of fluorophore dTomato-expressing *Coprinopsis cinerea* co-inoculated with *Bacillus subtilis* NCIB 3610 in a microfluidic device, resulting in hyphal cell collapse and the loss of fluorescence^19^. In the future, live-cell imaging with the presented device could be exploited further in combination with Raman spectroscopy as an emerging, non-invasive means to detect CWDE proteins around the cells involved in mycoparasitic interactions^30,31^. Certainly, the measured GFP signal within the *C. rosea* hyphal network that colonised the diamond segment anterior to the interaction zone after the initial mycoparasitic attack, suggests the presence of active transport of cytoplasm from the fungal prey along the hyphal network of its predator. This finding corresponds with the results of Schmieder, et al.^21^, which provide evidence for the long-distance transport of cytoplasmic solutes and signals within so-called ‘trunk’ hyphae of *C. cinerea,* utilising a confined microenvironment. Future investigations are needed to understand the biological processes underlying our observation and correlate the detection of GFP with a possible transfer of nutrients. For example, the double labelling with radioactive phosphorus isotopes ^32^P and ^33^P showed the transfer of phosphorus from attacked cells of *Rhizoctonia solani* into the developing mycelium of the mycoparasite *Arthrobotrys oligospora*^32^. The excellent control over the sampling time and the selection of sampling areas using a microfluidic device will strongly enhance the knowledge on cytoplasmic translocation and the spatial distribution of genetic responses during mycoparasitism. In fungal–nematode interactions, the use of a microfluidic device to obtain samples from interaction zones has shed new light on localised transcriptomic responses by a fungus under attack^20^.

The versatility of the FFI device was also demonstrated by investigating the interaction between *F. graminearum* and *T. rossicum*, a soil-dwelling fungus having the potential for bacteria to utilise its hyphae as a “fungal highway” within non-saturated environments^33^. A reliable and reproducible system, able to produce microenvironments that are not fully water-saturated, is a basic requirement to study this interesting phenomenon, as non-motile bacteria would strongly depend on the fungal hyphae to advance through the microchannels^34^. In the current study, fungal hyphae were found to draw liquid films into the FFI device repeatedly, which corroborates the fact that soil fungi are capable of redistributing water along gradients in water potential through their hyphal networks^35^. Furthermore, the growth of *T. rossicum* was inhibited in the presence of *F. graminearum*, suggesting the production of volatile or diffusible molecules by the pathogen with strong activity against *T. rossicum*. Lutz, et al.^36^ have reported the repressing effect of the important mycotoxin deoxynivalenol (DON) that has antimicrobial properties^37,38^, in the interaction with *T. atroviride* P1 strain, resulting in reduced expression of hydrolytic enzyme genes involved in antagonistic activity and saprophytic competition. However, no growth-inhibiting effects of DON were found in competitive assays on maize leaf tissue^39^. Certainly, the underlying mechanisms in the growth inhibition of *T. rossicum* by pathogenic *F. graminearum*, as well as the role of passive water transport by fungal hyphae in the migration of soil bacteria, present exciting questions for future investigations.

In conclusion, we expect numerous new research opportunities. Importantly, this study has demonstrated the high versatility of the FFI device, in terms of reproducible hyphal growth under various conditions and different combinations of fungal partnerships. Furthermore, the integrated control channels provide an option to differentiate between localised and systemic effects, which has previously proven to be valuable in studies of bacterial–fungal^40^ and fungal–nematode^21^ interactions using microfluidic devices. The possible addition of other rapidly advancing applications in the field of microfluidics, such as the detection of mycotoxins, could further expand the capabilities of our platform to better understand the trigger of toxin production during the interaction between various fungal species^41^. Naturally, the opportunity to simultaneously obtain qualitative and quantitative information on fungal growth inhibition at the cellular level could aid the discovery of new fungal BCAs against a wide range of pathogenic fungi.

## Methods

### Strains and culture conditions

Transformed wild-type (wt) strains of *Fusarium graminearum* Schwabe (teleomorph: *Gibberella zeae* [Schwein] Petch), the European strain 8/1-wt-GFP and the US-American strain PH1-dsRed, were employed in this study^42^. The constitutive expression of fluorophores was achieved by a fusion construct of the reporter gene *eGFP* and *dsRed*, respectively, with the glycerol-3-phosphate dehydrogenase promoter (*gpdA*) of *Aspergillus nidulans*^43^. Transformation by plasmid-mediated homologous integration facilitated the expression^44,45^. The antagonistic fungus *Clonostachys rosea* strain 016 was previously identified as a BCA against *F. graminearum*^25,46,47^. The fungus *Trichoderma rossicum* strain NEU135 was isolated from soil and showed positive interaction with soil-dwelling bacteria by facilitating their migration on hyphae^48^. Active cultures were maintained on oatmeal agar (20 g oatmeal and 15 g agar l^−1^ dH_2_O) for *F. graminearum* and *C. rosea* or on malt extract agar (12 g malt extract and 15 g agar l^−1^ dH_2_O) for *T. rossicum*. The strains were cultivated at either: (i) 18 ± 1 °C under a 12 h light (near visible ultraviolet light; black light blue tubular fluorescent lamps; 365 nm) (Osram, Germany)/12 h dark rhythm (*F. graminearum*) or (ii) 25 ± 1 °C in the dark (*C. rosea*, *T. rossicum*). All fungal isolates were stored on agar plugs (Ø 5 mm) at −70 °C in 50% glycerol solution.

### Microfluidic device fabrication

The design of the FFI device was constructed in AutoCAD Mechanical 2011 (Autodesk) and used to create a mylar® film photolithography mask (Micro Lithography Services Ltd., UK). The protocol for microfluidic device fabrication, including the manufacturing of the master mould, was previously described^19,49^. PDMS was formulated using a 10:1 ratio of base to curing agent (Sylgard 184, Dow Corning, USA) before degassing the mixture for 1 h under vacuum in a desiccator. The PDMS was poured onto the master mould, cured overnight at 70 °C, carefully removed from the mould and then cut into slabs. Subsequently, the two inlets for the fungal inocula were punched with a precision cutter (Ø 4.75 mm; Syneo, USA). After washing and drying the PDMS slabs, they were bonded onto glass-bottomed Petri dishes (Ø dish 50 mm, Ø glass 40 mm, glass thickness 0.17 mm; Fluorodish, World Precision Instruments, Germany) using a Zepto plasma cleaner (Diener Electronic, Germany; vacuum pressure 0.75 mbar, power 50%, coating time 1 min). Care was taken to bring the activated PDMS surface accommodating the embossed microchannels into direct contact with the glass. For experiments conducted under water-saturated and nutrient-rich conditions, the devices were filled immediately after bonding with autoclaved PDB (4 g potato starch and 20 g dextrose l^−1^ dH_2_O) (Difco, Becton Dickinson and Company, France) by pipetting 30 µl into each inlet (60 µl total). Subsequently, the devices were sterilised under UV light (254 nm) for 30 min. Nutrient-deficient devices were sterilised but not filled with liquid medium. In addition, 200 µl of sterile water was introduced into the perimeter of each Petri dish to maintain a high humidity upon closure of the lid.

### Inoculation of the FFI devices

For each of the three different FFIs, the experiments were set up separately: *F. graminearum* 8/1-wt-GFP vs. *C. rosea* 016, *F. graminearum* PH1-dsRed vs. *C. rosea* 016, and *F. graminearum* PH1-dsRed vs. *T. rossicum* NEU135. For each experiment, the fungal cultures were sub-cultured after one activating round of cultivation on their respective agar media. To inoculate the FFI device, agar plugs containing fungal mycelium (Ø 4 mm) were cut from the peripheral growth zone of 3–4-day-old cultures using a cork borer, ensuring that the growing hyphal front was left unharmed. Subsequently, the agar plug inocula were placed with the mycelium side facing the bottom of the fungal inoculation inlets and care was taken to orientate the hyphal tips in the direction of the microfluidic channels. The Petri dish was closed, sealed with two layers of laboratory film (Parafilm, Bremis Company Inc., USA) and the fungal species were incubated at 25 ± 1 °C in the dark. Controls included ‘self-interactions’ (*C. rosea* 016 vs. *C. rosea* 016) and ‘no interaction’ (fungal inoculum on one side was replaced with the respective medium). Each experiment was conducted three times, with two replicates tested in each experimental run. A detailed depiction of the experimental format is provided in Supplementary Fig. 9.

### Confrontation on agar plates

Petri dishes (Ø 9 cm) containing potato dextrose agar (4 g potato starch, 20 g dextrose and 15 g agar l^−1^ dH_2_O) (Difco, Becton Dickinson and Company, France) were inoculated with two plugs (Ø 4 mm) of fungal mycelium at a distance of 4.5 cm to each another and subsequently incubated at 25 ± 1 °C in the dark (Supplementary Fig. 10). The different combinations between each fungal partnership tested, including the controls, were set up for *C. rosea* 016 and *T. rossicum* NEU135 independently of each other. The radial growth of the fungal colony was quantified by measuring the distance (mm) using an electronic calliper (Tesa Technology Inc., Switzerland) in the direction towards the opposing side as described in Schöneberg, et al.^25^. The confrontation experiment was conducted twice with three replicates tested in each experimental run.

### Imaging

Pictures of the FFI device at defined time intervals for time-lapse experiments were acquired with an inverted microscope (Eclipse Ti-U, Nikon, Switzerland), equipped with an air immersed ×10/0.3 NA (numerical aperture) Plan Fluor objective (Nikon, Switzerland) and connected to a Retiga R1 CCD camera (Qimaging, Canada). A Prior scan III motorised stage (Prior Scientific, UK), mounted within a dark and temperature-controlled incubator (Okolab, Italy) at 25 °C, was used to acquire large images (covering 14 × 8 fields of view) with the NIS-Elements Advanced Research imaging software (Nikon, Switzerland). A 20% overlap was defined in order for the software to automatically stitch the images together and to form image montages. The autofocus software used the bright-field channel to determine the z-plane with 2 µm steps over a range of 20 µm. To acquire fluorescence images, a high-power light emitting diode (LED) light engine (LedHUB, Omicron-Laserage Laserprodukte GmbH, Germany) was used as the source of excitation. Two LEDs, with peak wavelengths of ~470 nm and ~505–600 nm, were employed to excite *F. graminearum* strains expressing GFP and dsRed, respectively. Subsequently, the following filters were used for each fluorophore, respectively: (i) band pass filters (Omicron-Laserage Laserprodukte GmbH; 465 nm and 495 nm), (ii) beam splitters (AHF Analysentechnik AG, Germany; 495 nm and 565 nm) and (iii) emission filters (AHF Analysentechnik AG; 525/50 nm and 605/70 nm).

### Image analysis and quantification

Images were processed and analysed using Fiji^50^. For the FFI experiments, the free hand line and measurement tools were used to determine the growth distance by measuring the length of the leading hyphae in pixels (px). This was defined as the hyphal tip that had advanced the furthest within each microfluidic channel at the time of acquisition. The growth distance was then determined by taking a tangent line to this leading hyphal tip, i.e. perpendicular to the direction of fungal growth, and measuring the distance from the beginning of the microchannel to this tangent line (Supplementary Fig. 11). The measurements within the individual microchannels were subsequently averaged (*n* = 2) and converted into micrometres (1 px = 0.64 µm) to perform statistical analyses. The leading hyphae of *C. rosea* 016 and *T. rossicum* NEU135 were measured using the phase contrast images, whereas the fluorescence microscopy images were used to measure *F. graminearum* growth. Fluorescence intensities (mean grey values) were measured at each time point of the time-lapse experiment with 10 min intervals between image acquisitions according to Schmieder, et al.^21^. The Fiji tool ‘rectangular’ was used to create a region of interest (ROI), which was placed exactly on the hypha of interest (covering the complete diameter of the hypha). To determine the relative fluorescence intensity, the value of the absolute fluorescence intensity within the hypha was divided by the absolute fluorescence intensity of the corresponding control channel. To measure the changes in the microchannel fluorescence intensity over time, the ROI was placed next to the hypha within the microchannel and measured. The relative fluorescence intensity was determined as described above. All measurements were performed six times on the same device and the respective average values were calculated. The relative fluorescence values in arbitrary units were plotted as a function of time.

### Statistics and reproducibility

All experiments were reproduced as indicated in the methods above. Data were arranged in Microsoft Excel (2016) and analysed in R Studio version 1.1.463^51^, running on R version 3.3.3^52^. Plots and figures were created using the ggplot2 package of R^53^ and Inkscape, version 0.92.2 for Mac OS (https://www.inkscape.org). All statistical analyses were performed using data of six independent replicates from each experiment (*n* = 6).

The comparisons between the control channels within the FFI device for different fungal interaction types, i.e. FFI, self-interaction and no interaction, were performed using linear mixed effects regression analysis with the R package lme4^54^. Diagnostic residual plots and Q-Q plots showed that the assumptions for linear modelling were met. Analysis of variance was carried out on the response variable ‘growth distance (µm)’ with the categorical variables ‘fungus’, ‘interaction type’ and their interaction as predicting factors. The significance level was set to *α* = 0.05. The time (hpi), experimental run and replicate within each experiment were set as random effects to account for variation of the response variable.

To synthesise the effects of FFI on hyphal growth, the relative growth rate comparing the growth of a given fungus before and after contact with the counteracting fungus was calculated according to Equation (1). For FFI experiments, the time span of 24–48 hpi and 48–72 hpi were defined as the pre hyphal interaction and post hyphal interaction period, respectively. For the confrontation on agar plates, the time span of 0–3 days post inoculation (dpi) and 3–7 dpi were defined in an analogous manner. Statistical comparison between the interacting fungi were performed using *t* test (data with normal distribution) or the Mann–Whitney *U* test (non-parametric test) with the significance level set to *α* = 0.05.

Equation (1), used to calculate the relative growth in FFI experiments:

$$Relative\,growth\,rate = \frac{{Growth\,{{rate}}\,post\,hyphal\,interaction\,({\mathrm{\mu }}m\,t^{ - 1})}}{{Growth\,rate\,pre\,hyphal\,interaction\,({\mathrm{\mu }}m\,t^{ - 1})}}$$

### Reporting summary

Further information on research design is available in the Nature Research Reporting Summary linked to this article.

## Supplementary information

Supplementary Information

Description of Additional Supplementary Files

Supplementary Movie 1

Supplementary Movie 2

Supplementary Movie 3

Reporting Summary

## Data Availability

Data sets acquired in this study are available for download at Zenodo 10.5281/zenodo.4421499^55^. These data sets contain the raw data from the quantification of fungal growth and fluorescence in FFI experiments, the measurement of hyphal diameters from microscope images as well as the processed movies of the FFI. In addition, all relevant data are available from the corresponding authors upon request.
