# Supplementary material for: A versatile microfluidic platform measures hyphal interactions between Fusarium graminearum and Clonostachys rosea in real-time
Source: Commun Biol. 2021 Feb 26;4:262. doi: 10.1038/s42003-021-01767-1 (PMC7910300; doi:10.1038/s42003-021-01767-1)
Supplement: Supplementary file 1 — Supplementary Information [file 42003_2021_1767_MOESM1_ESM.pdf]

Supplementary Information

A versatile microfluidic platform measures hyphal interactions between *Fusarium graminearum* and *Clonostachys rosea* in real-time

Authors:

Alejandro Gimeno ‡ , Claire E. Stanley ‡ *, Zacharie Ngamenie, Ming-Hui Hsung, Florian Walder, Stefanie S. Schmieder, Saskia Bindschedler, Pilar Junier, Beat Keller and Susanne Vogelgsang*

‡ These authors contributed equally

* Corresponding authors

Supplementary figures

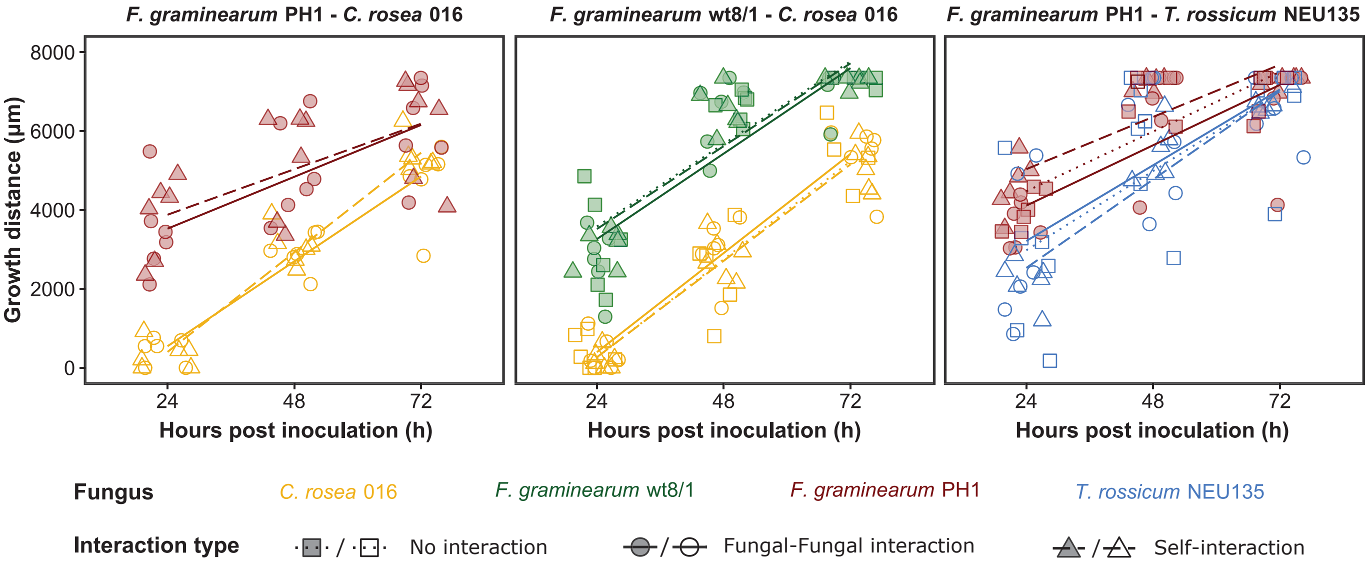

Supplementary Fig. 1 | Growth of *Fusarium graminearum* 8/1-wt-GFP, *F. graminearum* PH1-dsRed, *Clonostachys rosea* 016 and *Trichoderma rossicum* NEU135 in the control channels of the fungal-fungal interaction device. The control channel was analysed by plotting the hours post inoculation against the growth distance within the control channel for each fungus in the different interactions, i. e. no interaction (■), the interaction between *C. rosea* or *T. rossicum* vs. *F. graminearum* (●) and the self-interaction (▲). Overplotting of the data points was eliminated by adding a small amount of random variation to the horizontal location of each point using the jitter function in R (ggplot2 package). The comparison between the control channels for different interactions, were performed using linear mixed effects regression analysis. Analysis of variance (ANOVA) was carried out on the response variable 'growth distance (µm)' with the categorial variables 'fungus', 'interaction type' and their interaction as predicting factors. The time (hours post inoculation), experimental run and replicate within each experiment were set as random effects to account for variation of the response variable. The ANOVA results are reported in **Supplementary table 1**.

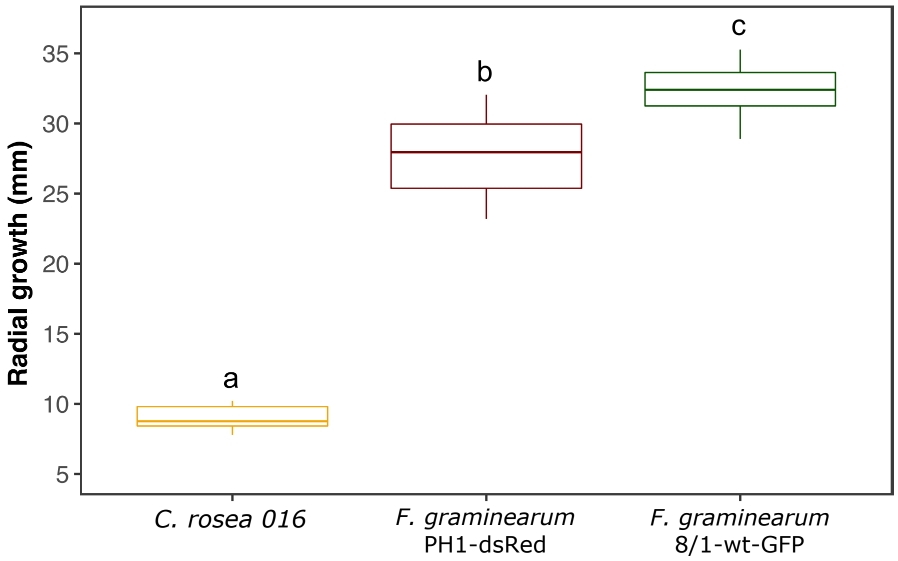

Supplementary Fig. 2 | Growth of *Fusarium graminearum* 8/1-wt-GFP, *F. graminearum* PH1-dsRed and *Clonostachys rosea* 016 on potato dextrose agar 72 hours post inoculation. Boxplots (n=6) with the median (line across box) and the 25th to 75th percentiles (box) of each distribution show the fungal species against their radial growth in millimeters on agar plate. Upper and lower whiskers represent the largest observation smaller or equal to the upper and lower percentile plus 1.5*interquartile range, respectively. One-way analysis of variance (ANOVA) was carried out between the categorial variable 'fungus'. Different letters above the boxes indicate significant differences according to post-hoc comparison using the Tukey test with the significance level set to 0.05.

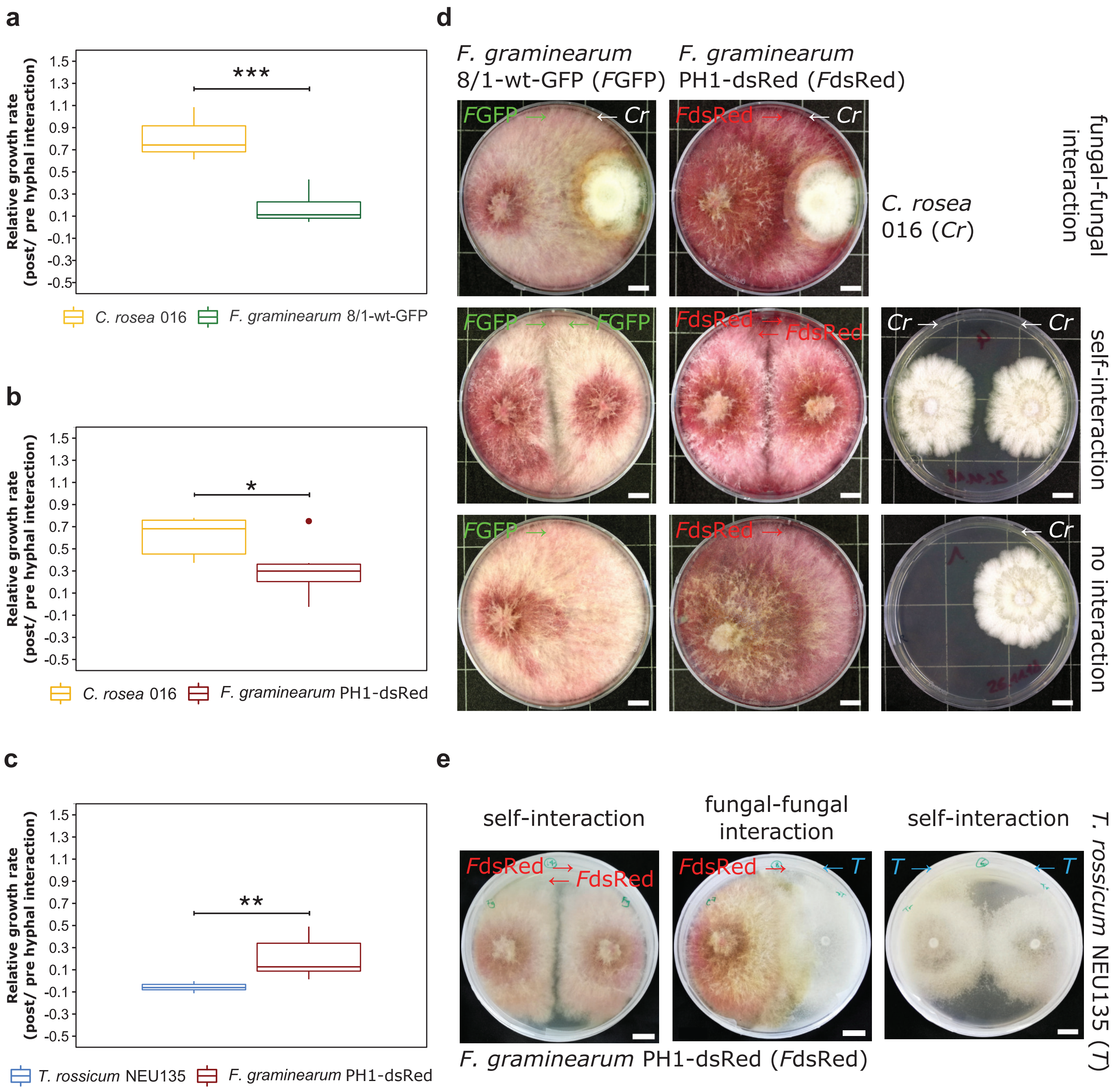

Supplementary Fig. 3 | Confrontation experiments on potato dextrose agar (PDA) plates. Boxplots (n=6) with the median (line across box) and the 25th and 75th percentiles of each distribution (box) show the relative growth rate (=growth rate 3 days post hyphal interaction / growth rate 3 days pre hyphal interaction) of (a) *Clonostachys rosea* 016 vs. *Fusarium graminearum* 8/1-wt-GFP, (b) *C. rosea* 016 vs. *F. graminearum* PH1-dsRed and (c) *Trichoderma rossicum* NEU135 vs. *F. graminearum* PH1-dsRed. Upper and lower whiskers represent the largest observation smaller or equal to the upper and lower percentile plus 1.5*interquartile range, respectively. Data points beyond the whisker range are plotted as dots. Significant differences according to the t-test (a) or the Mann-Whitney u-test (b, c) are indicated with asterisks for p-values < 0.001 (***), < 0.01 (**) and < 0.05 (*). (d) Photograph images of the confrontation experiments on PDA plates conducted with *C. rosea* 016 (*Cr*), *F. graminearum* 8/1-wt-GFP (*F*GFP) and *F. graminearum* PH1-dsRed (*F*dsRed) after 10 days as well as (e) *T. rossicum* NEU135 (*T*) and *F. graminearum* PH1-dsRed after seven days at 25° C in the dark. The arrows indicate the growth directions. Scale bar = 1 cm.

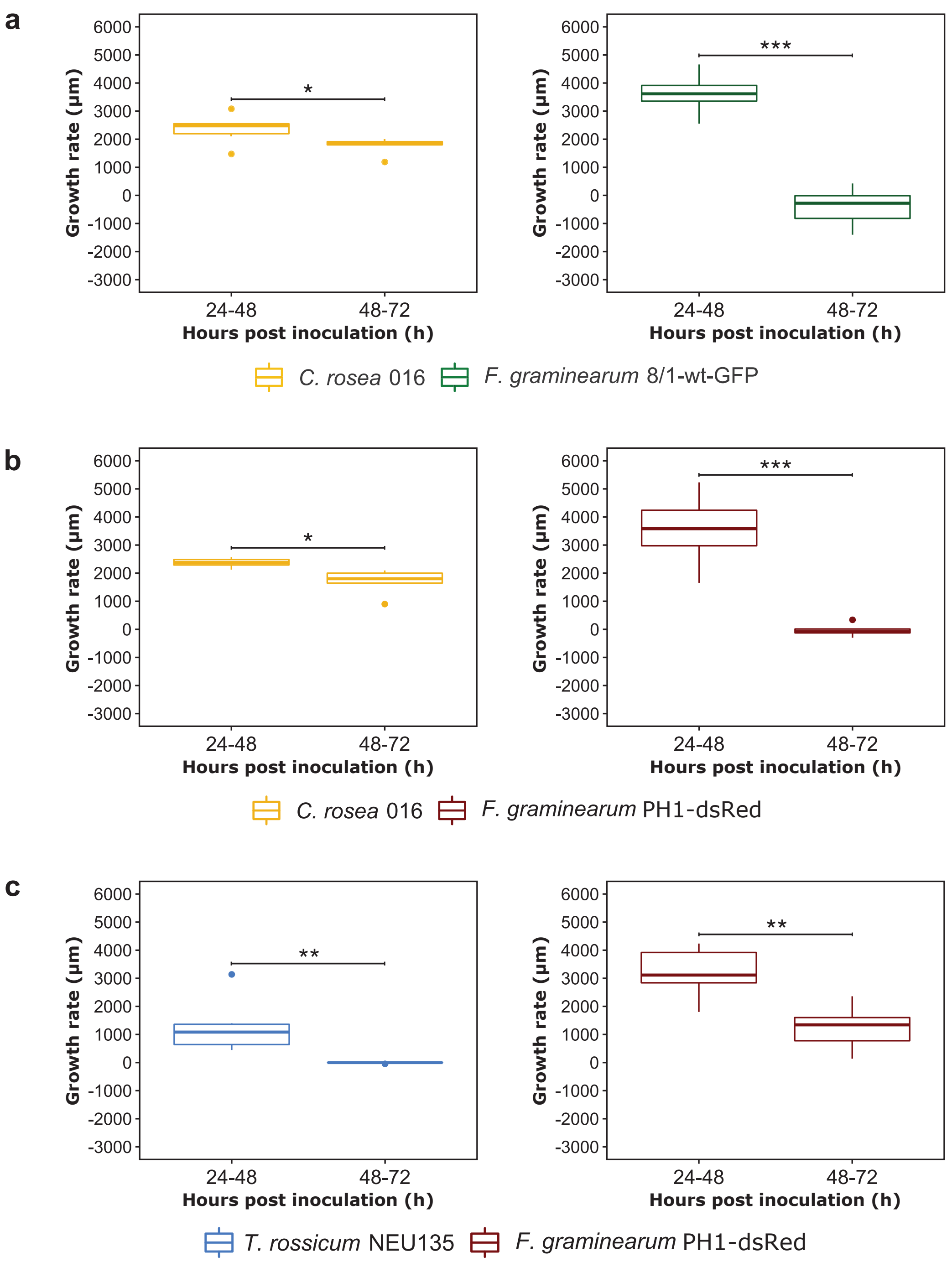

Supplementary Fig. 4 | Fungal-fungal interaction (FFI) device experiments. Boxplots (n=6) with the median (bar across box) and 25th to 75th percentiles (box) show the growth rates (µm) 24-48 hours post inoculation (hpi) (pre hyphal interaction period) and 48-72 hpi (post hyphal interaction period) for (a) *Clonostachys rosea* 016 vs. *Fusarium graminearum* 8/1-wt-GFP, (b) *C. rosea* 016 vs. *F. graminearum* PH1-dsRed in microchannels filled with potato dextrose broth and (c) *Trichoderma rossicum* NEU135 vs. *F. graminearum* PH1-dsRed in dry and nutrient-deficient microchannels. Upper and lower whiskers represent the largest observation smaller or equal to the upper and lower percentile plus 1.5*interquartile range, respectively. Data points beyond the whisker range are plotted as dots. Significant differences according to the t-test (*C. rosea*, *F. graminearum*) or the Mann-Whitney U test (*T. rossicum*) are indicated with asterisks for p values < 0.001 (***), < 0.01 (**) and < 0.05 (*).

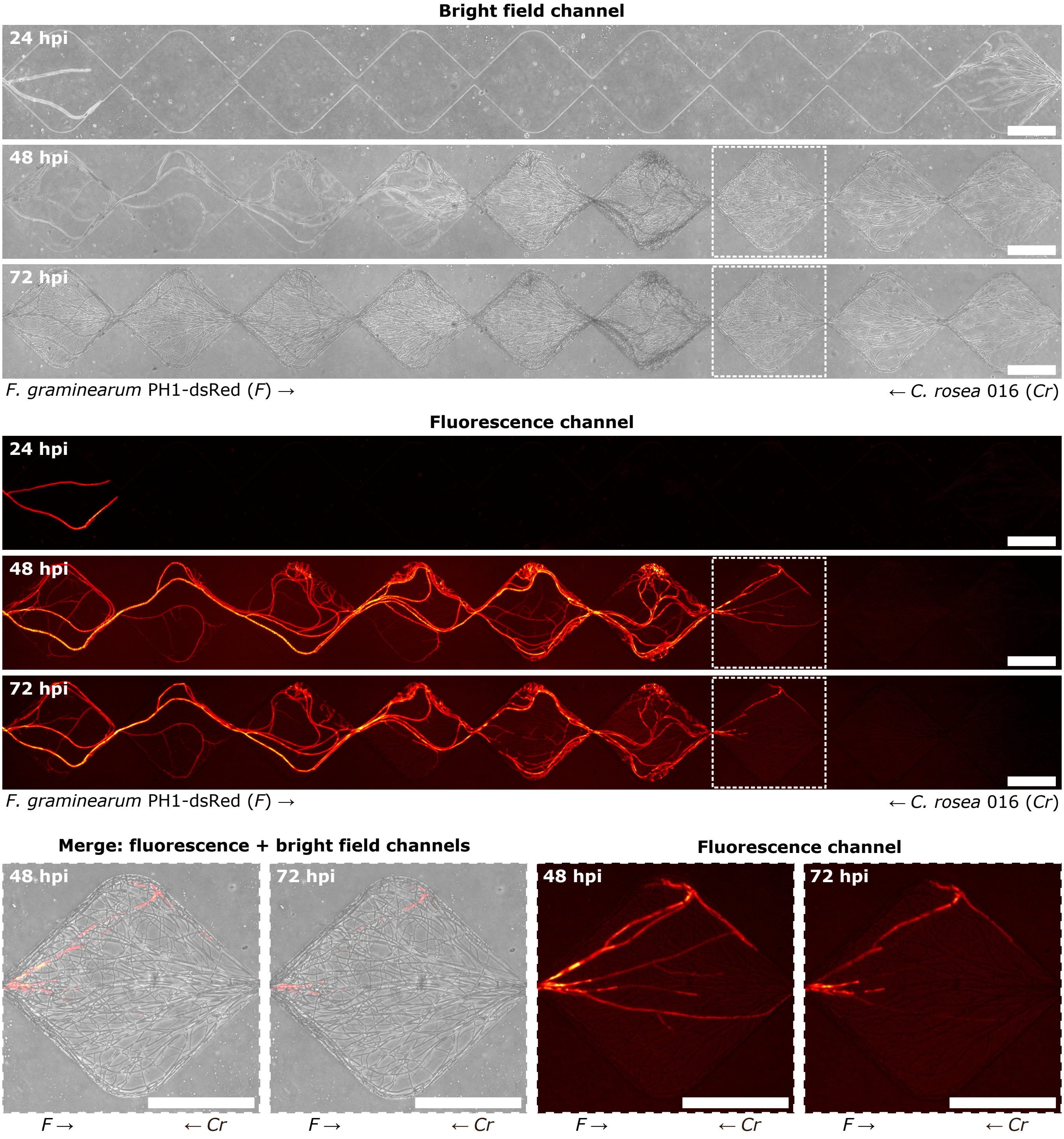

Supplementary Fig. 5 | Loss of fluorescence within hyphae of *Fusarium graminearim* PH1-dsRed in direct hyphal-hyphal contact with *Clonostachys rosea* 016 in the fungal-fungal interaction device. Microscopy image sections (9 diamond segments) taken from time-lapse experiments showing interaction between *C. rosea* 016 (*Cr*) vs. *F. graminearum* PH1-dsRed (*F*) in microchannels filled with potato dextrose broth after 24, 48 and 72 hours post inoculation (hpi). Dashed line squares refer to the enlarged sections showing the loss of fluorescence within *F. graminearum* hyphae after 72 hpi compared with 48 hpi. The arrows indicate the growth directions. Scale bar = 200 µm.

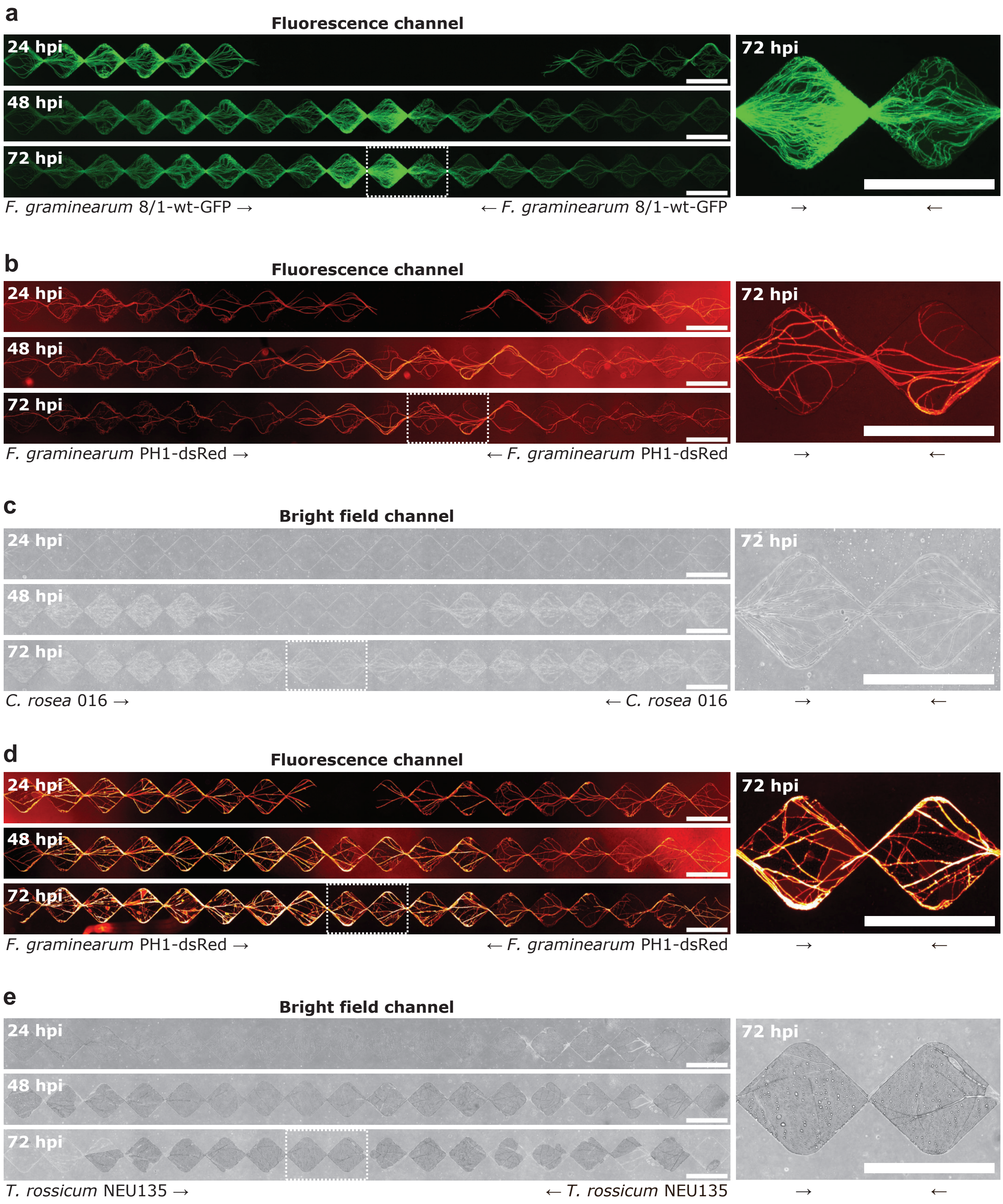

Supplementary Fig. 6 | Overview pictures of the interaction channel, 24, 48 and 72 hours post inoculation (hpi) in the self-interaction. (a) *Fusarium graminearum* 8/1-wt-GFP, (b) *F. graminearum* PH1-dsRed and (c) *Clonostachys rosea* 016 growing in the interaction channel filled with potato dextrose broth. (e) *F. graminearum* PH1-dsRed and (e) *Trichoderma rossicum* NEU135 growing in dry and nutrient-deficient interaction channel. Dashed line squares refer to the enlarged sections on the right side. The arrows indicate the growth directions. Scale bar = 500 µm.

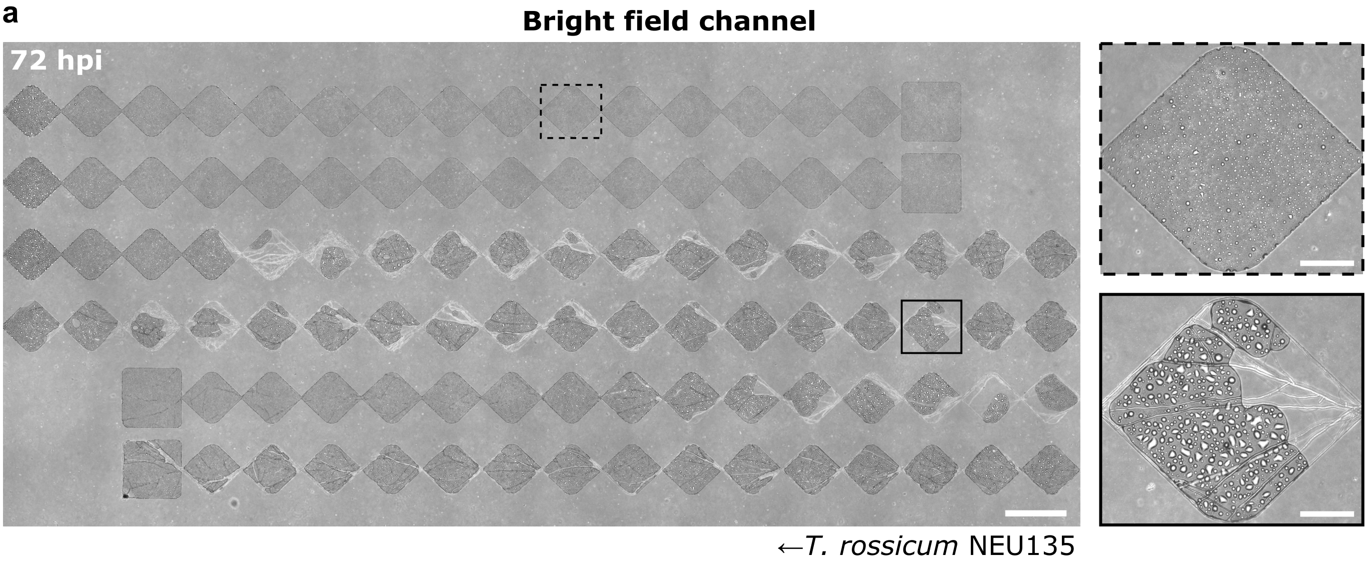

Supplementary Fig. 7 | Liquid films around the hyphae of *Trichoderma rossicum* NEU135 or *Fusarium graminearum* PH1-dsRed within the fungal-fungal interaction device. (a) Overview of phase contrast (bright field channel) microscopy images, showing the 'no interaction' setup with *T. rossicum* in dry microchannels 72 hours post inoculation (hpi). The empty control channels remained dry, while water film formation occured in colonised segments. (b) Overview of merged phase contrast and fluorescence microscopy images, showing the 'no interaction' setup with *F. graminearum* in dry microchannels 48 hpi. The empty control channels remained dry, while water film formation occured in colonised segments. Dashed and solid line squares refer to the enlarged sections on the right side. The arrows indicate the growth directions. Scale bars = 500 µm (overviews) and 100 µm (enlarged segments).

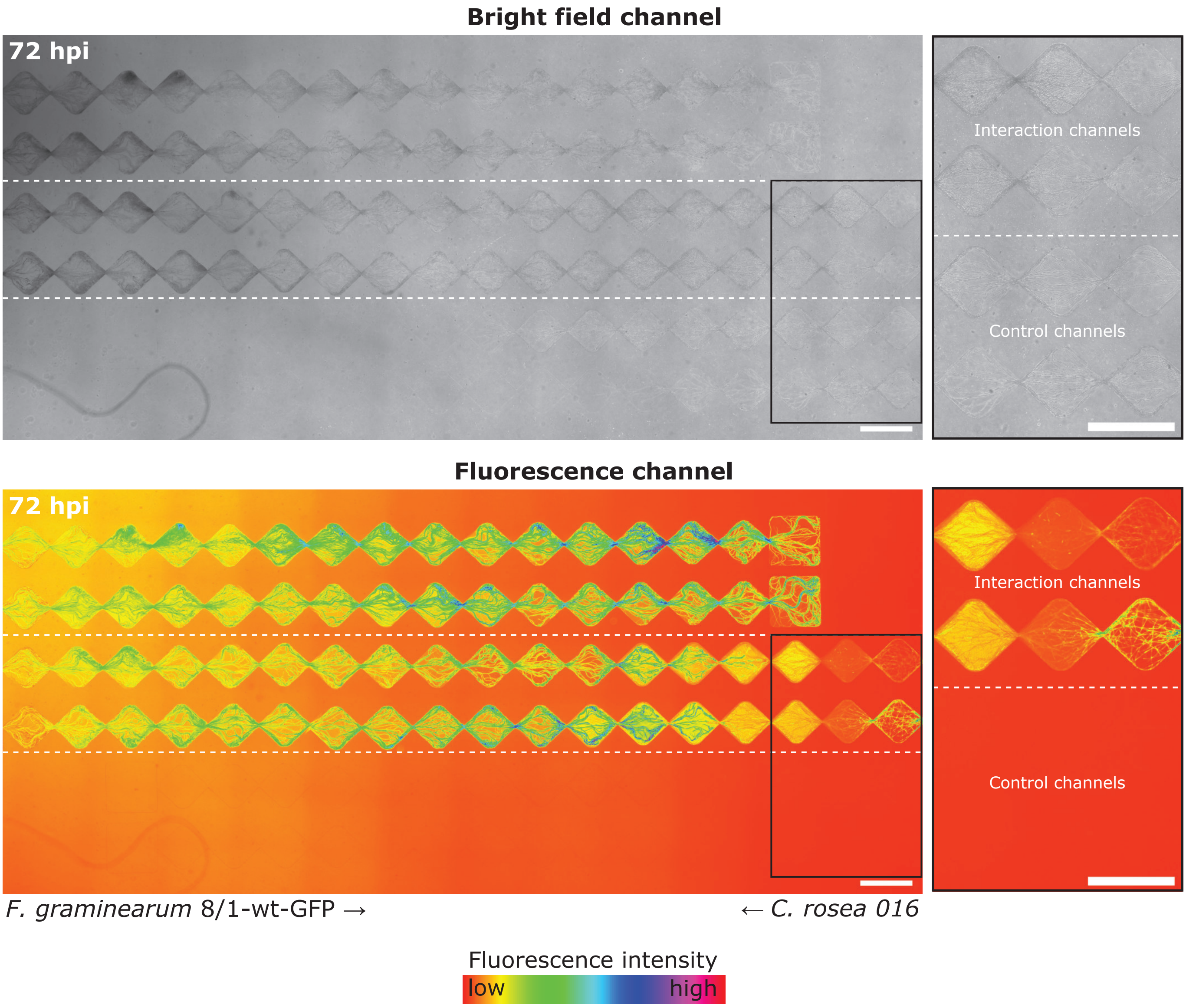

Supplementary Fig. 8 | *Clonostachys rosea* 016 antagonising *Fusarium graminearum* 8/1-wt-GFP within the fungal-fungal interaction device. Overview of phase contrast (bright field channel) and fluorescence microscopy images 72 hours post inoculation. The solid line squares refer to the enlarged sections on the right side. The lookup table "spectrum" (Fiji) was applied to improve the visibility of fluorescence intensity. The fluorescence microscopy shows detection of GFP fluorescence within the hyphal network of *C. rosea* that colonises the segment anterior to the fungal-fungal interaction zone. GFP fluorescence in *C. rosea* hyphae within the adjacent control channels was not detected. The arrows indicate the growth directions. Scale bar = 500 µm.

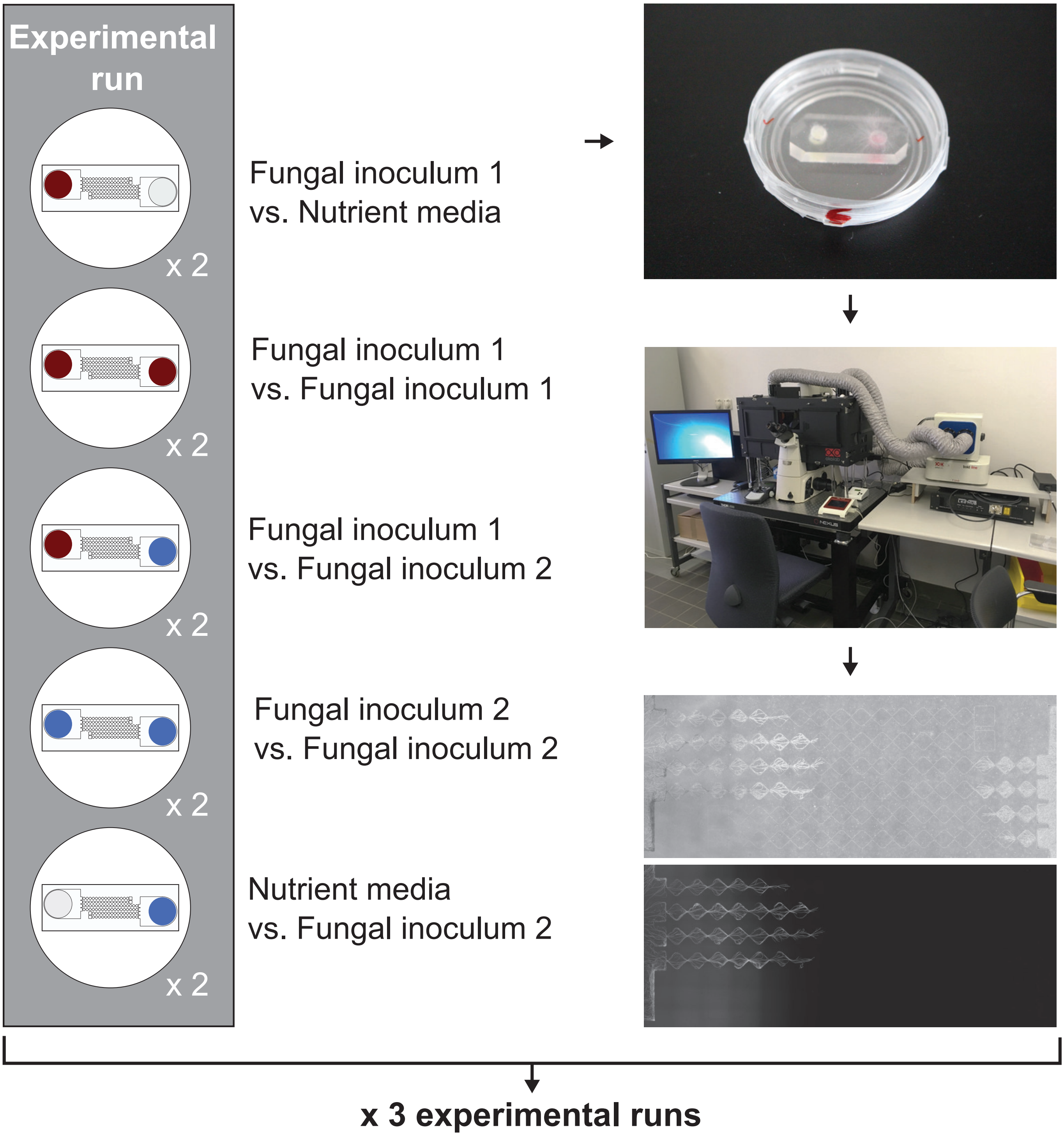

Supplementary Fig. 9 | Experimental format of the fungal-fungal interaction experiments. Within each experiment, *Fusarium graminearum* 8/1-wt-GFP vs. *Clonostachys rosea* 016, *F. graminearum* PH1-dsRed vs. *C. rosea* 016 (microchannels filled with potato dextrose broth) and *F. graminearum* PH1-dsRed vs. *Trichoderma rossicum* NEU135 (dry and nutrient-deficient microchannels), the different combinations between each fungal partnership were set up in two technical replicates per experimental run. Experiments were repeated three times and carried out at 25 ± 1 °C in the dark. Overview microscope images (bright field and fluorescence) were aquired within a dark and temperature controlled incubator (25 ± 1 ºC). *Note*: After the validation of the control channel in the experiment between *F. graminearum* 8/1-wt-GFP vs. *C. rosea* 016, the 'no interaction' devices, i.e. nutrient media vs. fungal inoculum', were omitted from the experiment *F. graminearum* PH1-dsRed vs. *C. rosea* 016.

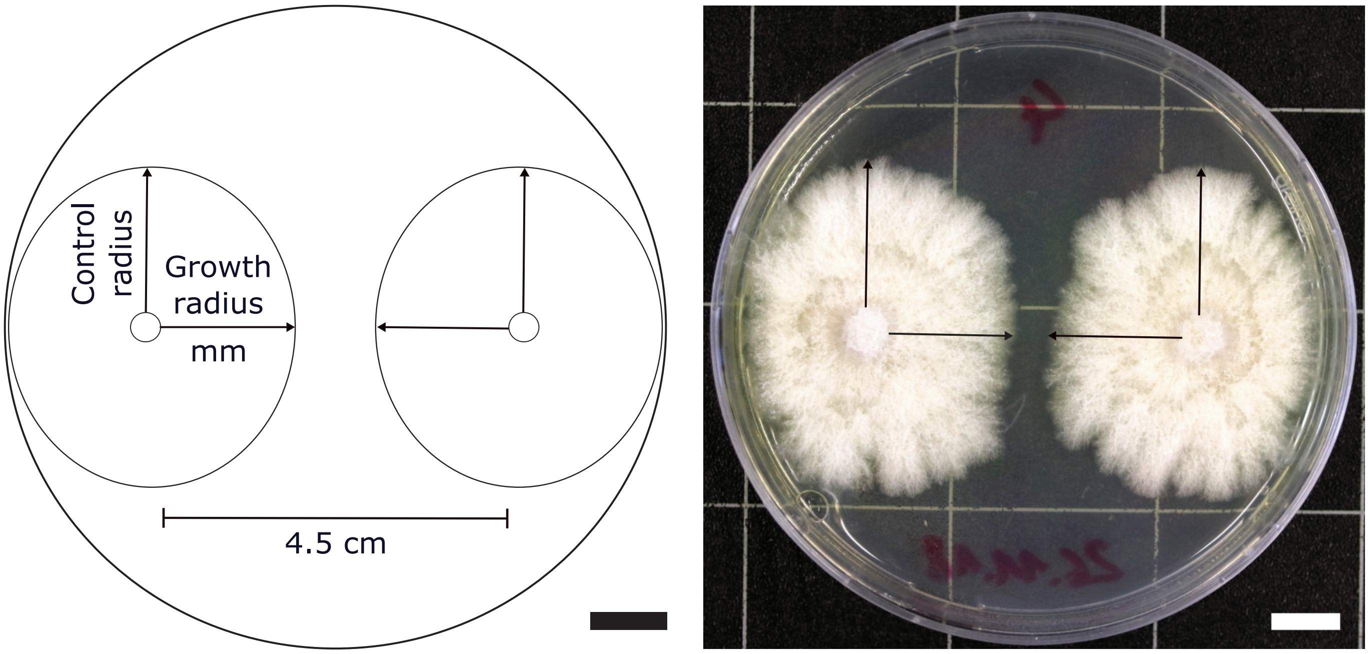

Supplementary Fig. 10 | Quantification of the radial growth of fungal colonies in the confrontation assay on agar plates. Left: schematic presentation; right: photographic image of an example (*Clonostachys rosea* 016). The distance towards the direction of the opposing colony or nutrient media was measured in a straight line from the fungal inoculum to the growing front of the colony. An additional control measurement was taken in perpendicular direction. Scale bar = 1 cm.

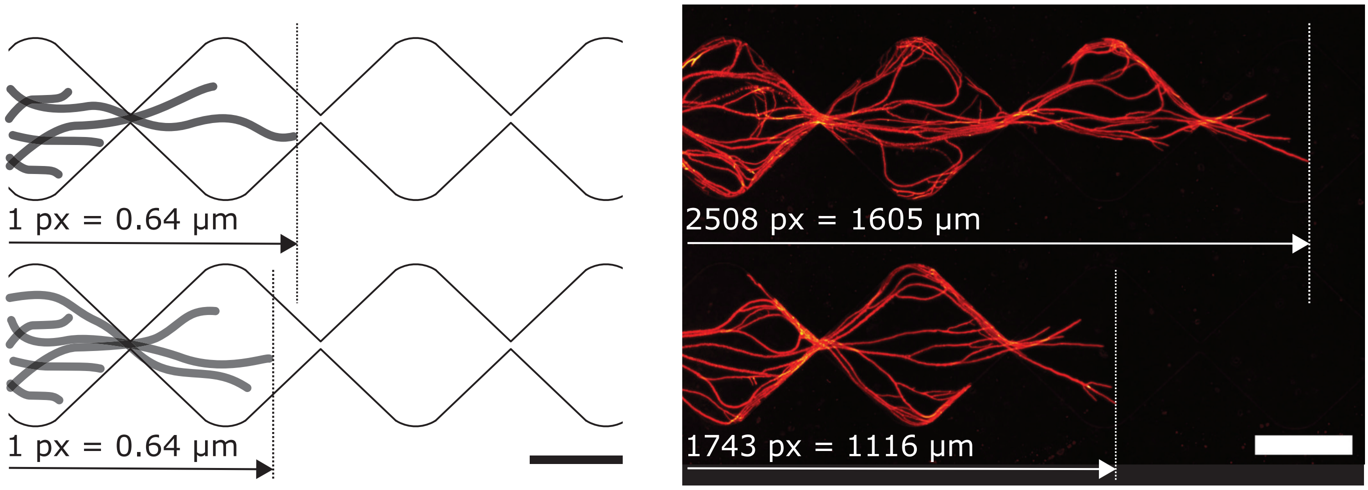

Supplementary Fig. 11 | Quantification of growth distance within microchannels of the fungal-fungal interaction device from microscopy images using ImageJ (Fiji). Left: schematic presentation; right: microscopic image of an example (*Fusarium graminearum* Ph1-dsRed). The free hand line and the measurement tools were used to determine the length (in pixels), of the leading hyphae within each channel by taking the tangent line to the hyphal tip that had advanced the furthest at the time of aquisition and measuring the distance from the beginning of the microchannel to this tangent line. The number of pixels was converted into µm (1 px = 0.64 µm). Scale bar = 250 µm.

Supplementary tables

Supplementary Table 1 | Results of the analyses of variance (ANOVAs) on the growth of *Fusarium graminearum* 8/1-wt-GFP, *F. graminearum* PH1-dsRed, *Clonostachys rosea* 016 and *Trichoderma rossicum* NEU135 in the control channels of the fungal-fungal interaction device. The comparison between the control channels within the FFI device for different fungal interaction types, i.e. fungal-fungal interaction, self-interaction and no interaction, were performed using linear mixed effects regression analysis. Diagnostic residual plots and Q-Q plots showed that the assumptions for linear modelling were met. ANOVA was carried out on the response variable 'growth distance (µm)' with the two categorical variables 'fungus' and 'interaction type', as well as their interaction, as predicting factors. The significance level was set to α = 0.05. The time (hours post inoculation), experimental run and replicate within each experiment were set as random effects to account for variation of the response variable. ns = not significant.

	F. graminearum PH1-dsRed – *C. rosea* 016			*F. graminearum* 8/1-wt-GFP – *C. rosea* 016			*F. graminearum* PH1-dsREd – *T. rossicum* NEU135		
	Df	*F* value	p value	*Df*	*F* value	p value	*Df*	*F* value	p value
Fungus	1	99.72	< 0.001	1	388.09	< 0.001	1	29.68	< 0.001
Interaction type	1	0.98	ns	2	0.02	ns	2	0.30	ns
Fungus × Interaction type	1	0.01	ns	2	0.95	ns	2	2.72	ns
