# Supplementary material for: A versatile microfluidic platform measures hyphal interactions between Fusarium graminearum and Clonostachys rosea in real-time
Source: Commun Biol. 2021 Feb 26;4:262. doi: 10.1038/s42003-021-01767-1 (PMC7910300; doi:10.1038/s42003-021-01767-1)
Supplement: Supplementary file 2 — Description of Additional Supplementary Files [file 42003_2021_1767_MOESM2_ESM.pdf]

Description of Additional Supplementary Files

File name: Supplementary Movie 1

Description: Liquid films around fungal hyphae of *Trichoderma rossicum* NEU135 and *Fusarium graminearum* PH1-dsRed within microchannels of the fungal-fungal interaction device. A time-lapse experiment was recorded over 26 hours with 2 h intervals between image acquisitions using phase contrast (bright field channel) and fluorescence microscopy. The bright field and fluorescence images were merged to create an overlay image. The movie shows liquid films around the fungal hyphae drawn into the microchannel and the progressive increase of the water content. Scale bar = 200 µm. Time stamp format = hh:mm.

File name: Supplementary Movie 2

Description: *Clonostachys rosea* 016 antagonising *Fusarium graminearum* 8/1-wt-GFP within the fungal-fungal interaction device. A time-lapse experiment was recorded over 19 hours and 30 minutes with 10 min intervals between image acquisitions using phase contrast (bright field channel) and fluorescence microscopy. The movie shows continued growth of *C. rosea* around the pathogen *F. graminearum* and the increasing loss of the green fluorescence protein (GFP) detection from hyphae of GFP-expressing *F. graminearum*. Note: On the bottom right side, the detection of GFP fluorescence in hyphae of *C. rosea* occurs concomitantly to the loss of GFP fluorescence in *F. graminearum* hyphae. Scale bar = 200 µm. Time stamp format = hh:mm.

File name: Supplementary Movie 3

Description: Detection of GFP fluorescence in hyphae of *Clonostachys rosea* 016. Region of interest from a time-lapse experiment recorded over 19 hours and 30 minutes with 10 min intervals between image acquisitions using phase contrast (bright field channel) and fluorescence microscopy. The movie shows the detection of GFP fluorescence within the interconnecting hyphal network of *C. rosea* that colonised the diamond segment anterior to the fungal-fungal interaction zone. The lookup table "spectrum" (Fiji) was applied to improve the visibility of the fluorescence intensity. The fluorescence intensity detected in these hyphae increased and decreased, before it finally increased again over time. Scale bar = 50 µm. Time stamp format = hh:mm.
