# Supplementary material for: A versatile microfluidic platform measures hyphal interactions between Fusarium graminearum and Clonostachys rosea in real-time
Source: Commun Biol. 2021 Feb 26;4:262. doi: 10.1038/s42003-021-01767-1 (PMC7910300; doi:10.1038/s42003-021-01767-1)
Supplement: Supplementary file 6 — Reporting Summary [file 42003_2021_1767_MOESM6_ESM.pdf]

natureresearch

Corresponding author(s): Susanne Vogelgsang
Claire Stanley

Last updated by author(s): 2020/07/28

Reporting Summary

Nature Research wishes to improve the reproducibility of the work that we publish. This form provides structure for consistency and transparency in reporting. For further information on Nature Research policies, seeAuthors & Referees and theEditorial Policy Checklist .

Statistics

For all statistical analyses, confirm that the following items are present in the figure legend, table legend, main text, or Methods section.

n/a	Confirmed	
☐	☒	The exact sample size (*n*) for each experimental group/condition, given as a discrete number and unit of measurement
☐	☒	A statement on whether measurements were taken from distinct samples or whether the same sample was measured repeatedly
☐	☒	The statistical test(s) used AND whether they are one- or two-sided<br>*Only common tests should be described solely by name; describe more complex techniques in the Methods section.*
☐	☒	A description of all covariates tested
☐	☒	A description of any assumptions or corrections, such as tests of normality and adjustment for multiple comparisons
☐	☒	A full description of the statistical parameters including central tendency (e.g. means) or other basic estimates (e.g. regression coefficient) AND variation (e.g. standard deviation) or associated estimates of uncertainty (e.g. confidence intervals)
☐	☒	For null hypothesis testing, the test statistic (e.g. *F*, *t*, *r*) with confidence intervals, effect sizes, degrees of freedom and *P* value noted<br>*Give P values as exact values whenever suitable.*
☒	☐	For Bayesian analysis, information on the choice of priors and Markov chain Monte Carlo settings
☒	☐	For hierarchical and complex designs, identification of the appropriate level for tests and full reporting of outcomes
☒	☐	Estimates of effect sizes (e.g. Cohen's *d*, Pearson's *r*), indicating how they were calculated

Our web collection on statistics for biologists contains articles on many of the points above.

Software and code

Policy information about availability of computer code

Data collection	Microscope image aquisition and processing:<br>NIS-Elements Advanced Research imaging software (Nikon, Switzerland)<br>Fiji (ImageJ) opensource image processing software
Data analysis	Statistical analysis:<br>R Studio version 1.1.46348, running on R version 3.3.349

For manuscripts utilizing custom algorithms or software that are central to the research but not yet described in published literature, software must be made available to editors/reviewers. We strongly encourage code deposition in a community repository (e.g. GitHub). See the Nature Research guidelines for submitting code & software for further information.

Data

Policy information about availability of data

All manuscripts must include a data availability statement. This statement should provide the following information, where applicable:

- Accession codes, unique identifiers, or web links for publicly available datasets
- A list of figures that have associated raw data
- A description of any restrictions on data availability

Data sets containing the raw data acquired during this study will be made available via Zenodo. The following Data Availability Statement will appear in any finalised version of the manuscript:
"Data sets acquired in this study are available for download at Zenodo https://doi.org/10.5281/zenodo.3685957. This data set contains the raw data from the quantification of fungal growth and fluorescence in FFI experiments, the measurement of hyphal diameters from microscope images as well as the processed movies of the FFIs."

Field-specific reporting

Please select the one below that is the best fit for your research. If you are not sure, read the appropriate sections before making your selection.

- [x] Life sciences
- [ ] Behavioural & social sciences
- [ ] Ecological, evolutionary & environmental sciences

For a reference copy of the document with all sections, see nature.com/documents/nr-reporting-summary-flat.pdf

Life sciences study design

All studies must disclose on these points even when the disclosure is negative.

Sample size

For each group that was compared statistically, the sample size was 6.
In the experiments with the microfluidic devices, the samples were collected in 3 experimental rounds with two replicates in each round.
In the experiments on agar plates, the samples were collected in 2 experimental rounds with three three replicates in each round.

Data exclusions

No data were excluded from the analyses.

Replication

In experiments with the microfluidic devices, the experiments were performed 3 times with two replicates in each round.
In experiments on agar plates, the experiments were performed 2 times with three replicates in each round.

Randomization

The microfluidic devices and agar plates were fully randomised upon incubation and analysis with no sequential order.

Blinding

Blinding was not relevant to this study. Data collection was performed based on standardised image aquisition.

Reporting for specific materials, systems and methods

We require information from authors about some types of materials, experimental systems and methods used in many studies. Here, indicate whether each material, system or method listed is relevant to your study. If you are not sure if a list item applies to your research, read the appropriate section before selecting a response.

Materials & experimental systems

n/a	Involved in the study	
x		Antibodies
x		Eukaryotic cell lines
x		Palaeontology
	x	Animals and other organisms
x		Human research participants
x		Clinical data

Methods

n/a	Involved in the study	
x		ChIP-seq
x		Flow cytometry
x		MRI-based neuroimaging

Animals and other organisms

Policy information about studies involving animals; ARRIVE guidelines recommended for reporting animal research

Laboratory animals

This study did not involve laboratory animals.

Wild animals

This study did not involve wild animals.

Field-collected samples

This study did not involve samples collected from the field.

Ethics oversight

No ethical approval or guidance was required because there are no ethical implications associated with the organisms used in this study.

Note that full information on the approval of the study protocol must also be provided in the manuscript.
